# Research Ethics Boards: The Protection of Human Subjects

**DOI:** 10.1371/journal.pmed.0030472

**Published:** 2006-10-31

**Authors:** Valia S Lestou, Nancy Ondrusek, Morris A Blajchman

Compelling arguments, documentation, and published data have always been the basis of scholarly examination, discussion, and reasoning. Rhetorical catch phrases, biased ideologies, and anecdotal reports, when used to prove erroneous and often unrelated points, are creating condemnation and polarization instead of awareness on the important issues of research ethics boards.

The statement that “they are in a client–provider business relationship” as expressed in the article by Lemmens and Elliott [1] implies a direct financial relationship between sponsors and those reviewing their research protocols. This is incorrect. Since we cannot speak on behalf of other for-profit Institutional Review Boards (IRBs), we will describe how our Canadian for-profit IRB functions. The owners of the company and those involved in the running of the business are not IRB members and have no voting privileges. IRB members are external consultants who are paid by the company providing the review services for the time spent in reviewing a protocol and providing an expert ruling regarding the ethics and science of a particular study. IRB members have no business interests in the company (e.g., partnership or profit-sharing) and no vested interest in the approval or non-approval of a specific study. It is worth noting that members of academic or governmental not-for-profit IRBs are often paid for the ethical review services they provide, in recognition of the time and skill required to carry out their duties.

Respected, well-organized, and accredited IRBs (for-profit and not-for-profit) are not an enterprise of “five people”. This is merely the minimal regulatory requirement, which we consistently exceed in our meetings. IRBs are complex bodies of highly qualified professional experts from various disciplines, such as physicians, pharmacists, scientists, lawyers, philosophers, ethicists, and lay persons. They are multicultural, balanced in gender, diverse in social background, and dedicated to the protection of research subjects (which remains the main mandate for all IRBs). Often, for-profit IRB members are serving or have served as IRB members on academic or governmental not-for-profit IRBs. Is it expected that the work of these individuals is ethical when they are volunteers, and unethical when they are remunerated for their expertise?

Rather than fixate on the concept that members of IRBs are paid, we should concentrate on the real challenges regarding IRBs (for-profit and not-for-profit) and try to propose some meaningful insight. Ethics in biomedical research is a serious and complex affair with huge societal implications. Expert advisors that are paid or not paid are not the problem that IRBs face today. It is rather the rapidly growing complexity of biomedical research that confronts IRBs, together with the lack of a timely transfer of knowledge, often due to insufficient resources, the lack of continuing education, and the lack of sufficient and appropriate member expertise. IRBs are expected to function as multifaceted oversight bodies and assume many duties, including ethics consultation, education, peer reviewing, adverse events reporting, publication policies, and the review of financial conflicts of interest.

The attention should be focused on whether IRBs are performing to the highest ethical standards and conforming to existing regulations rather than if their members are remunerated or not. Singling out the issue of payment distracts from sincere discussion on how to improve and ensure high-quality reviews of biomedical research and maintain the protection of the human subjects involved.
